# Block Work and Continuous Gum

**Published:** 1855-01

**Authors:** 


### ARTICLE VII.

### *Block Work and Continuous Gum.* By A. A. B.

WE have been long contemplating a short notice of these beautiful methods of mounting artificial teeth; the difficulty of coming to a decided preference, has restrained our remarks, but finding that a multiplicity of letters and questions are before us unanswered, upon this subject, we may be allowed by our general reader, an expression of experience for the benefit of those seeking our knowledge. We do not think that the first class pieces of block work for beauty, in or out of the mouth, can be possibly excelled, provided that when designed for wear, they have been fortunately worked and skillfully adapted. The art of making block teeth is one of such peculiar nicety, as to constitute in its perfection, a talent requiring the highest order of genius, with the most steadfast and persevering industry. Nor will we ever retract from this high standard, to which is unfortunately devoted, a meagre portion of the talent of the profession, from a false division of the mechanical and operative departments being founded upon an imaginative superior excellence required in the one over the other, whereas it will frequently be found, that the good operator has no aptitude for the delicate manipulations of the carver, and we boldly affirm, that the skillful block-workman is always susceptible of becoming the highest order of operator. From this fact, a method somewhat freed from the great art required in block teeth had been sought after for a long time, and at last found by two gentlemen, Drs. Hunter and Allen, to whom the credit is due of having made the most valuable accession to mechanical dentistry during any period of its past history, and styled by them *continuous gum work.* In passing, we cannot but regret that the serious difficulty still exists between these gentlemen, as to priority of introduction; for we are assured that the whole profession, as well as the immense number of patients, recipients of their ingenious production, will yield to them a heartfelt gratitude and a praise that will live long after they shall cease to be numbered among the living.

This work, although equally beautiful with block teeth, does not require the same order of talent to produce a similar result, in fact it is block work simplified, and from its greater strength, as well as greater facility of repairing when broken, improved. Thus an individual can readily learn to construct it in one-third the time that he will require to enable him to carve and mount blocks, provided he possesses the native art necessary to produce good carving. We believe that every one having any mechanical tact, can be taught to construct the continuous gum, after an experience necessary to guard against accidents occurring in construction, but only a few who can become equally skillful in the working of blocks. Not that the evidence of superior workmanship is wanting in different pieces, but that a novice or an individual whose aptitude for mechanism may not be striking, yet could be instructed so as to execute a tolerable set of teeth in this way, who would never carve and mount a passable set of block teeth. As in single teeth there can be easily traced the difference of a master hand over the indifferent workman, more especially in the color and form perhaps than from any other particular, for the identical body and gum will give an entirely different result, both as regards the color and shape of the gum, according to the degree and duration of heat it has been exposed to. The platina plates forming the base for the compound, are only required to be of the usual thickness of gold plates for block teeth, or rather thinner than is required for single teeth, from the necessity of rimming inside and out, stiffening it sufficient for all practical purposes. This must be well done with strips of the metal about the same thickness as the plate itself, and varying in width from $\frac{1}{8}$ to $\frac{1}{4}$ inch, as the beauty of the piece demands. After the outer rim is adjusted and soldered with pure gold, the teeth are arranged and held in their proper places for backing, by pouring around them equal parts of plaster and fine asbestos; this is dried and the whole piece cut so as to enter the muffle of your furnace with ease. You now proceed to back the teeth, in doing which, several points must be attended to, for the purpose of securing strength. Each backing should come down in easy contact with the plate, so that the gold will be sure to fasten them. The backings should be of double the thickness of the plate, and soldered down in the muffle of the furnace, the heat of which must approach whiteness.

It is not necessary that the pins should be riveted, but merely closed upon the backing, so as to retain it to be securely fastened by the gold solder. Each backing should be wide enough to come in contact with its neighbor, so that the whole should be soldered together, as one continuous sheet of backing. After this has been done, you must proceed to adjust another band which should be so fitted as to touch the backing of each tooth and the plate at every point, and thus well soldered, will give you a result incredibly strong; at any rate, sufficient to resist all customary liabilities.

The next step after cleansing the teeth and plate, is to put the base or body forming the gum around and between the teeth. This is used about the consistency of thick cream, and made to run into every interstice which may exist in or about the teeth and plate, and partly dried from time to time, so as to prevent its running over the surface of either, and finally pressed and carried into the shape of the gum, as required by the prominence of the alveolus, or by the contour of the face, allowing for the thickness of the gum which is yet to be put on. It must now be crused or biscuited sufficiently hard to handle safely, and to permit of a quick absorption of the gum, which must be applied with a brush or knife, at about the same consistency as the body or base, securing it equally thick all over, which must be about $\frac{1}{16}$ of an inch, owing entirely to the color or strength of the composition, as also to the amount of titanium or coloring matter put in the body. Great care must now be used so that it should dry very slowly, subjecting it to the lowest heat, and gradually increasing up to the fusing point; this will prevent cracking and spawling, and permit that shrinkage in the composition which gives to the gum a very natural appearance, depressing, as it does, between each tooth; but if the heat be too suddenly applied, this is utterly prevented, and much cracking and blistering certainly follows.

In selecting the teeth they should all if possible be made to rest upon the plate as in mounting single teeth, but this is not absolutely essential, as good base is equally strong as the teeth and even more so than most of those now used for this purpose.

If they touch the plate, and the fusing is not produced too rapidly, the gum will present the reticulated appearance at once, so beautiful and natural.

It most generally becomes necessary to return the piece in the furnace, owing to the cracks and fissures left principally around the teeth, and where the plate and composition connects. These must be filled with the liquid gum coloring and dried; then filled again until solid and even; the piece must now again be subjected to heat, but perhaps less carefully than before, as there is less danger of spawling, from the dryness of the plate and body. It will now most probably be found completed, and should be dressed down, and polished for the mouth; this is best done with fine sand paper, but there will be found little particles of dust of the base, upon the plate, or rim that cannot be thus removed, and can be done so, only by the use of pulverized silex, and a stick, or what is better corundum slab. One difficulty, which although slight, and whose effects can be easily overcome, yet at times gives some trouble and much delay, is from the enamel of the teeth adhering to the composition of plaster and asbestos, which is placed around the points to prevent their moving from their positions during the time the base is fusing, and the teeth without being protected would be drawn out of their proper connection. It often happens that the enamel of the teeth being composed simply of spar, and even mixed with glass, fuses at as low a temperature as the continuous base, and thus unites with the plaster and asbestos placed to hold the teeth, which must be polished from their surfaces. This will be obviated in a great degree by the following precaution. The plaster and asbestos is formed in a circle of similar diameter with the teeth, pressed solid, and about $\frac{3}{8}$ of an inch thick, and whilst still soft, the points of the teeth forced downwards and backwards in it, $\frac{1}{8}$ of an inch, thus they will be strongly supported within, and to a slight depth in front, effectually preventing the movement of any of them.

It requires great experience to regulate the heat of the furnace with exactness, and will perhaps be found to present the greatest obstacle to young beginners, who must be very cautious not to rely upon guessing as to the time requisite, but keep a constant examination, as there can no possible harm result to a piece by withdrawing it from the furnace, examining it with a light, and returning it as soon as possible; let this be done twenty times if necessary, as one minute too long will prove fatal to the most beautiful worked piece.

In cases of blistering, the entire protuberances must be ground out, and filled up, if very deep with the base, and then covered with gum, thus preventing the disagreeable effect of blotches, which must ensue if filled up alone with the gum color.

For repairing, this method surpasses all others for neatness and strength; the broken tooth is ground out, down to the plate, and a new one fitted within the aperture thus made; it is now backed, and perhaps with a double backing, so as to bring its surface on a line with the continuous rim; the base and gum is now flowed in and around the tooth, filling the space a little more than flush, to allow for shrinkage; the piece placed in plaster and asbestos, and the gold put on for soldering its backing to the rim and plate, the fluxing of the base and soldering being accomplished with one heat. Thus, if the piece has been shaken or broken by the injury in any other place, the whole fluxes again, and becomes as solid as when first completed, the place of repair is entirely concealed externally if skillfully done, the inner side only to be observed by the color of the gold used in soldering. There is little or no trouble about the shrinkage of the plate. Occasionally, when the base is required to be laid on heavy, and the heat applied at too high a temperature, the plate may be sprung a little, but is easily reduced by hammering or pressing to the plaster models. This feature alone is of much value, for the springing of gold plates always has been, and most likely ever will be, a point of much difficulty to overcome. It will also be found of considerable advantage in the construction of all temporary sets, by merely using a rim of platina, covering only the alveolus, which has been stamped upon the silver plate, allowing the platina to run up over the ridge for the purpose of carrying the gum. The process is precisely the same as for a full platina plate, only with the difference of soldering the rim and silver plate together when completed, with hard or soft solder; if the latter, it should be applied only in the centre of the ridge of the alveolus, leaving the space between the outer and inner edges of the platina rim, this space to be filled either with gutta percha, or what is better, equal parts of feldspar and sulphur, heated and forced in during partial fusion. This hasty description may perhaps represent a tedious process, but if so, every one who will attempt it, will be, after a little practice, happily deceived, for it undoubtedly is as speedy a method as can be adopted in the construction of any kind of artificial teeth, with the great advantage of being more desirable in every respect.

How different is it in block work, the time and care required in getting up your models, the preparation of the materials, the alterations of color, the carving, crusing, enameling and baking, are attended with so much that is doubtful and tedious, as to render it a process of much anxiety and weariness; then comes the grinding, adjusting, rimming, and soldering; all equally troublesome, and less certain and perfect in the end. The repairs in this work are, to the thorough workmen, the reconstruction of the whole piece, for it is rare indeed to find a piece of work creditable which has been repaired: to stop to draw a comparison between single teeth, and either of these two processes, but particularly with the former, is indeed loss of time and is in fact behind the age.

No dentist who is doing a practice which produces a reasonable income, should hesitate to acquire the means of working this admirable plan of constructing sets of teeth. It will pay him over and over again the first year, and lift him a good century forward.

